# Corneal and Ocular Aberrations: A Comparative Study of Hyperopic, Emmetropic, and Myopic Eyes

**DOI:** 10.18502/jovr.v21.18138

**Published:** 2026-02-16

**Authors:** Amir Golmakani, Negareh Yazdani, Jamshid Jamali, Asieh Ehsaei

**Affiliations:** ^1^Department of Optometry, School of Paramedical Sciences, Mashhad University of Medical Sciences, Mashhad, Iran; ^2^Refractive Errors Research Center, Mashhad University of Medical Sciences, Mashhad, Iran; ^3^Department of Biostatistics, School of Health, Mashhad University of Medical Sciences, Mashhad, Iran; ^4^Social Determinants of Health Research Center, Mashhad University of Medical Sciences, Mashhad, Iran

**Keywords:** Aberrations, Emmetropia, Hyperopia, Myopia, Zernike Coefficients

## Abstract

**Purpose:**

To investigate differences in corneal and ocular aberrations between hyperopic, emmetropic, and myopic eyes.

**Methods:**

This cross-sectional study included 84 eyes from 84 healthy participants (age range 18-35 years), who were equally divided into three refractive error groups of hyperopia, emmetropia, and myopia. Following comprehensive optometric examinations, corneal and ocular aberrations were measured using Pentacam and Zywave imaging systems, respectively. Aberrations were measured for a pupil diameter of 5 mm. Root-mean-square (RMS), Zernike coefficients, and compensation factor (CF) between total ocular and corneal aberrations were calculated for statistical analysis.

**Results:**

The mean spherical equivalent was +1.96 ± 1.64, –0.17 ± 0.19, and –2.46 ± 1.56 diopters in hyperopic (H), emmetropic (E), and myopic (M) eyes, respectively. The amount of corneal Zernike coefficients Z_4_^–2^ in the hyperopic eyes (H: 0.015 ± 0.040) was significantly higher than that of emmetropic eyes (E: –0.018 ± 0.054) and myopic eyes (M: –0.003 ± 0.038) with a *P-*value of 0.029. The RMS of corneal total coma (H: 0.228 ± 0.097, E: 0.183 ± 0.086, M: 0.164 ± 0.083, *P*-value: 0.009, respectively) and RMS of ocular fifth-order (H: 0.045 ± 0.018, E: 0.036 ± 0.020, M: 0.032 ± 0.016, *P*-value: 0.002) showed a significant difference between refractive error groups, with higher value in hyperopic eyes compared to the two other groups. There was no significant difference in CF for RMS and Zernike coefficients among the refractive error groups.

**Conclusion:**

Hyperopic eyes showed a higher RMS value for ocular fifth-order and corneal coma aberrations compared to the emmetropic and myopic eyes.

##  INTRODUCTION

Aberrations are a form of optical irregularity that play a vital role in retinal image quality and vision.^[1]^ Aberrations are classified as lower-order aberrations (LOAs) and higher-order aberrations (HOAs), both of which can be represented mathematically using Zernike polynomials.^[2]^ LOAs comprise first- and second-order aberrations including defocus and astigmatism, while HOAs include aberrations of third-order and higher^[2]^ such as coma, trefoil, and spherical aberrations, which are quantified in micrometers and can be expressed as a root-mean-square (RMS) value.^[3]^ It has been shown that aberrations are influenced by several factors, including ethnicity,^[3]^ pupil diameter,^[4]^ refractive error,^[5,6]^ corneal shape,^[7]^ and age.^[8]^

Recent advances in ocular imaging have expanded our ability to investigate the optical characteristics of the eye, surpassing traditional sphere and cylinder descriptions and allowing for a comprehensive assessment of ocular and corneal aberrations.^[2]^ Mapping HOAs enables a comprehensive evaluation of ocular optical characteristics that is beneficial across fields, including wavefront-guided refractive surgery,^[9]^ wavefront-guided contact lens prescription, and post-surgical management.^[10,11]^ Numerous studies have explored the relationship between ocular and corneal aberrations and the refractive status of the eye.^[5,6][12][13]^ While some studies have reported that myopic eyes exhibit significantly higher levels of ocular RMS of HOAs,^[5]^ others attribute these higher values to hyperopic eyes.^[6]^ Moreover, internal compensation in HOAs may reduce overall HOA levels in both adults and children.^[14,15]^

Developing our understanding of corneal and ocular aberrations across different refractive errors will have significant implications for various surgical fields, enabling new approaches to refractive correction and improving visual performance.^[16,17,18]^ This study evaluates both ocular and corneal aberrations and calculates the compensation factor (CF) between them. Thereby, we acquire a more comprehensive understanding of the optical system across various refractive statuses, including hyperopia, emmetropia, and myopia. Assessing CF enhances our understanding of how internal optics compensate for corneal aberrations and is particularly relevant to advancing refractive surgery planning and wavefront-guided optical correction.

##  METHODS

### Participants

This cross-sectional study included 84 eyes from 84 healthy individuals admitted to Khatam-al-Anbia Eye Hospital. The study was approved by the Research Ethics Committee of Mashhad University of Medical Sciences and followed the tenets of the Declaration of Helsinki. The study procedure was explained to the participants, and they entered the study after submitting written informed consent.

All participants, who belonged to the same ethnic background, underwent a thorough ocular examination, including slit lamp evaluation of the anterior and posterior segments, and were recruited if their right eye fulfilled the inclusion criteria. These criteria were: being 18-35 years of age, spherical equivalent refractive error between –6.00 and +6.00 diopters (D) with astigmatism < 1 D, best-corrected visual acuity of 0.00 LogMAR (Logarithm of the Minimum Angle of Resolution) or better, and no contact lens use for at least 2 weeks before examinations. On the other hand, the exclusion criteria included a history of any ocular surgery, corneal scar, dry eye disease, or pupil size < 5 millimeters (mm) that could interfere with measurement procedures. The Topcon Auto-keratorefractometer 8900 (Topcon Corp., Tokyo, Japan) was used to determine the average of three consecutive refraction measurements for each eye. Ocular refraction was reported as the mean spherical equivalent (MSE) and categorized into three groups: emmetropia (E; MSE between +0.25 D and - 0.25 D), myopia (M; MSE ≤ - 0.5 D), and hyperopia (H; MSE ≥ +0.5 D).^[19]^

### Ocular Examinations 

Corneal and ocular aberrations were assessed using Pentacam AXL (Oculus Optikgeräte GmbH, Wetzlar, Germany) and Zywave II (Bausch & Lomb, Rochester, NY, USA), respectively.

**Figure 1:**
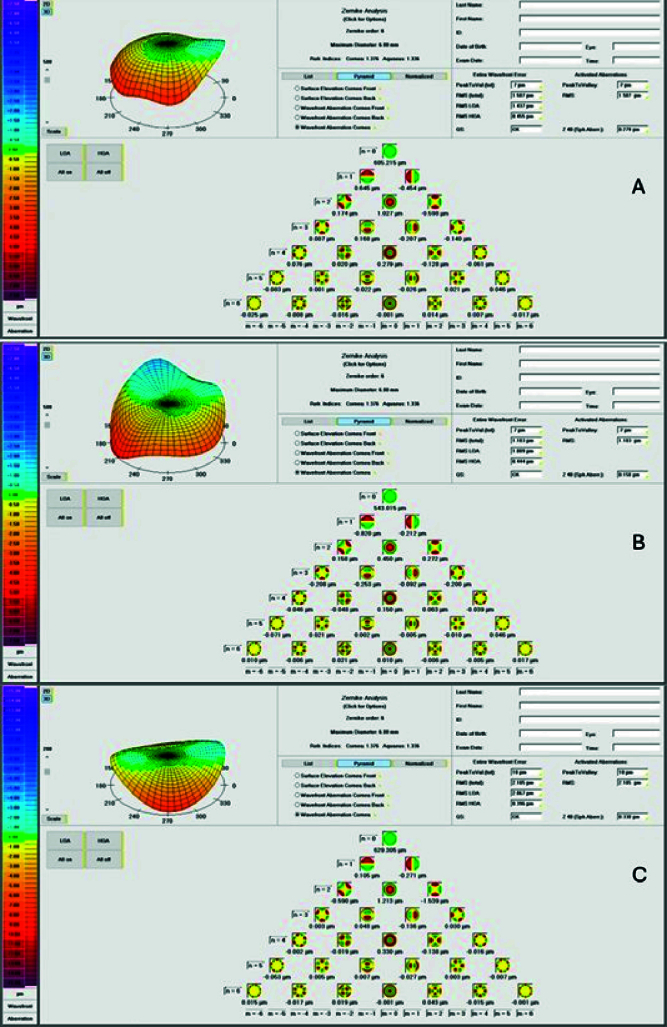
Corneal Zernike analysis presented by Pentacam for hyperopic (A), myopic (B), and emmetropic (C) eyes. Higher-order aberrations (HOAs) were presented up to the sixth order.

During Pentacam imaging, participants were instructed to blink twice before each measurement and then fixate on the target. Subsequently, the Scheimpflug camera was adjusted with the joystick to achieve optimal quality scoring, denoted as “OK” before image acquisition. For ocular aberrometry using Zywave II, the reference cross was centered at the pupil surface, and focus was adjusted until the edge between the pupil and iris was clearly defined. Participants were then prompted to blink once and maintain both eyes fully open during this phase. Aberrometry measurements were conducted under scotopic conditions and analyzed at a pupil diameter of 5 mm.^[20]^ The Pentacam AXL provided corneal aberration data classified into standard Zernike categories, including astigmatism (Z_2_^–2^, Z_2_^2^, Z_4_^–2^, Z_4_^2^), defocus (Z_2_^0^), trefoil (Z_3_^–3^, Z_3_^3^, Z_5_^–3^, Z_5_^3^), coma (Z_3_^–1^, Z_3_^1^, Z_5_^–1^, Z_5_^1^), tetrafoil (Z_4_^–4^, Z_4_^4^), spherical (Z_4_^0^), and pentafoil (Z_5_^–5^, Z_5_^5^) [Figure 1]. The Zywave II measured these same Zernike aberrations, but for the entire ocular system. The disparity between corneal and ocular aberrations was assessed by computing the RMS of the third to fifth orders. The RMS was calculated using the Zernike coefficients according to the single indexing system, as depicted in the equation below:^[21]^


$$RMS=\sqrt{\sum_{i=1}^{j}{\left(C_{i}\right)}^{2}}$$

To evaluate the degree of internal ocular compensation for RMS and Zernike coefficients, we calculated the CF using the following equation:^[14]^


$${CF}_{RMS}=1-\frac{{RMS}_{ocular}}{{RMS}_{cornea}}$$

A value of CF for RMS can be between 0 and 1, where 0 shows no compensation and 1 shows total compensation. The CF for the Zernike coefficients is determined using the following equation:^[14]^


$${CF}_{RMS}=1-\frac{{C_{n}^{m}}_{ocular}}{{C_{n}^{m}}_{cornea}}$$

One possible outcome of these corneal CF calculations is that small corneal HOAs ( ≤ 0.05 µm) may produce an inaccurately large negative value. As a result, the CFs for the RMS values and the specific Zernike coefficients related to the fifth order were excluded from analysis.

### Statistical Analysis

Statistical analyses were conducted using SPSS version 25.0 (IBM Corp., Armonk, NY, USA). The normal distribution of the data was assessed using the Kolmogorov-Smirnov normality test. Statistical analysis was performed using one-way analysis of variance (ANOVA), followed by a Bonferroni multiple comparisons test. The Pearson correlation coefficient was used to assess correlations among variables, and *P* < 0.05 was considered statistically significant.

##  RESULTS

We analyzed a total of 84 eyes from 84 participants with a mean age of 27.73 ± 4.62 years. Notably, significant differences in pupil diameter were observed among refractive groups, with emmetropic eyes exhibiting the largest pupil diameter and hyperopic eyes the smallest (E: 6.55 ± 0.63 mm; H: 6.14 ± 0.57 mm; M: 6.26 ± 0.90 mm; *P* = 0.004). The mean Q-value did not differ significantly among the three refractive groups (E: - 0.37 ± 0.12; H: - 0.31 ± 0.09; M: - 0.35 ± 0.11; *P* = 0.116). Angle kappa was significantly greater in hyperopic eyes (0.26 ± 0.13º) compared to emmetropic eyes (0.23 ± 0.10º) and myopic eyes (0.15 ± 0.07º; *P* < 0.001). Table 1 presents participants' demographic data and a comprehensive summary of the study.

**Table 1:** Demographic and baseline ocular characteristics of participants in the emmetropic, hyperopic, and myopic groups. Values are presented as mean ± standard deviations unless otherwise indicated.

	**Total**	**Emmetropia**	**Hyperopia**	**Myopia**
Age (Y)	27.73 ± 4.62	27.21 ± 4.53	29.54 ± 4.82	26.43 ± 4.05
MSE (D)	–0.22 ± 1.57	–0.17 ± 0.19	+1.96 ± 1.64	–2.46 ± 1.56
Gender (F/M)	52 / 32	19 / 9	12 / 16	20 / 8
Q-value	–0.34 ± 0.11	–0.37 ± 0.12	–0.31 ± 0.09	–0.35 ± 0.11
Angle Kappa	0.21 ± 0.11	0.23 ± 0.10	0.26 ± 0.13	0.15 ± 0.07
Y, year; MSE, mean spherical equivalent; D, diopter; F, female; M, male.				

### Corneal and Ocular Lower-order Aberrations (LOAs)

No statistically significant differences in corneal LOAs were observed among the three refractive groups. However, significant differences were identified, with Z_2_^–2^ and Z_2_^2^ values demonstrating an increased positive shift in myopic eyes compared to the other groups, while Z_2_^0^ represented a more positive value in hyperopic eyes. The quantification of corneal LOAs revealed the following values: Z_2_^–2^ (E: 0.085 ± 0.314 µm; H: 0.067 ± 0.284 µm; M: 0.109 ± 0.264 µm; *P* = 0.439), Z_2_^0^ (E: 0.444 ± 0.362 µm; H: 0.572 ± 0.396 µm; M: 0.465 ± 0.313 µm; *P* = 0.522), and Z_2_^2^ (E: –0.636 ± 0.435 µm; H: –0.552 ± 0.410 µm; M: –0.524 ± 0.410 µm; *P* = 0.363).

Statistical analysis revealed a significant difference in ocular LOA for the Z_2_^0^ value among the three refractive groups, with a positive value in hyperopic eyes compared to myopic and emmetropic eyes (E: –0.299 ± 0.333 µm; H: 1.636 ± 1.723 µm; M: –2.089 ± 1.517 µm; *P* < 0.001). In contrast, Z_2_^–2^ (E: –0.047 ± 0.182; H: –0.018 ± 0.240; M: –0.011 ± 0.218; *P* = 0.781), and Z_2_^2^ (E: 0.054 ± 0.249; H: 0.062 ± 0.307; M: 0.060 ± 0.287; *P* = 0.533) did not exhibit a statistically significant difference across the refractive error groups.

### Corneal High-order Aberrations

**Figure 2:**
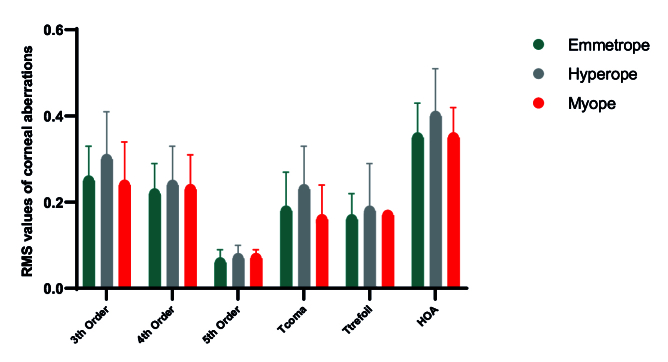
Mean RMS values of corneal aberrations, including the 3 ${}^{\text{rd}}$ -, 4 ${}^{\text{th}}$ -, and 5 ${}^{\text{th}}$ -order aberrations, total coma (T-coma), total trefoil (T-trefoil), and higher-order aberrations (HOAs), in three refractive groups: emmetropes (green), hyperopes (grey), and myopes (red). Data are presented as means, and error bars represent the standard error of the mean.

Table 2 presents the distribution of individual corneal Zernike coefficients from the third to fifth orders along with their respective RMS values for the three refractive error groups. It is noteworthy that corneal aberrations showed little or no difference across hyperopic, emmetropic, and myopic groups. The only exception was Z_4_^–2^, which showed a statistically significant difference across refractive groups (*P* = 0.02), with the highest value in hyperopic eyes (0.015 ± 0.040), followed by myopic eyes (–0.003 ± 0.038) and emmetropic eyes (–0.018 ± 0.054). Figure 2 shows the RMS values of corneal aberrations, revealing a significant difference in the RMS of T-coma (*P* = 0.009) among the three refractive groups, with the highest and lowest values in hyperopic and myopic eyes, respectively.

### Ocular High-order Aberrations 

**Figure 3:**
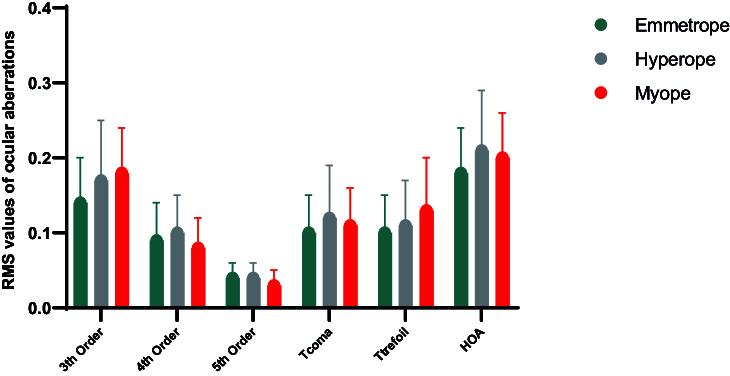
Mean RMS values of ocular aberrations, including the 3 ${}^{\text{rd}}$ -, 4 ${}^{\text{th}}$ -, and 5 ${}^{\text{th}}$ -order aberrations, total coma (T-coma), total trefoil (T-trefoil), and higher-order aberrations (HOAs), in three refractive groups: emmetropes (green), hyperopes (grey), and myopes (red). Data are presented as means, and error bars represent the standard error of the mean.

Table 3 further outlines the distribution of individual ocular Zernike coefficients, ranging from the third to fifth orders, along with the RMS values for the three groups. The mean RMS values of ocular HOAs in myopic, hyperopic, and emmetropic eyes were 0.199 ± 0.065, 0.210 ± 0.077, and 0.178 ± 0.062, respectively. Figure 3 presents ocular RMS values across the three refractive categories, demonstrating a statistically significant difference in the fifth-order RMS (*P* = 0.002) among these groups. Notably, hyperopic eyes exhibited the highest RMS values, while myopic eyes displayed the lowest.

### Compensation Factor (CF)

**Figure 4:**
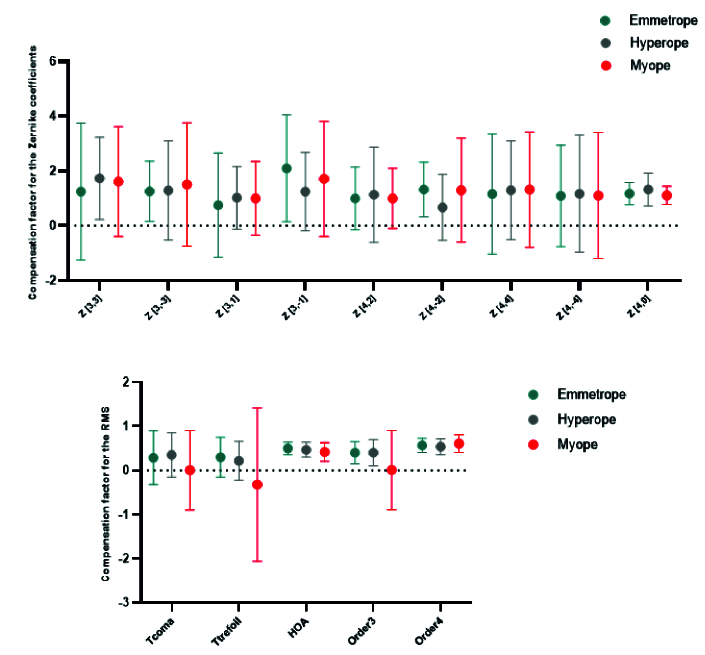
Mean compensation factor of individual Zernike coefficients (upper graph) and mean compensation factor of RMS values (lower graph) across three refractive groups: emmetropes (green), hyperopes (grey), and myopes (red). Values are presented as means, with error bars indicating the standard error of the mean.

Figure 4 shows the mean CF values for the Zernike coefficients and RMS, indicating no significant difference between the refractive error groups.

### Correlation Between RMS and MSE

No correlation was observed between ocular RMS of HOAs and MSE in emmetropia (*r* = 0.033, *P* = 0.866), hyperopia (*r* = 0.123, *P* = 0.534), and myopia (*r* = –0.115, *P* = 0.560). Similarly, there was no correlation between corneal RMS of HOAs and MSE in emmetropia (*r* = 0.103, *P* = 0.603), hyperopia (*r* = 0.214, *P *= 0.273), and myopia (*r* = 0.087, *P *= 0.659). Also, there was no correlation between the CF of RMS and MSE in the three study groups.

**Table 2:** Corneal aberration coefficients and RMS for refractive error groups

**Zernike term**		**Myopia**	**Hyperopia**	**Emmetropia**	***P*** **-value**	**Pairwise comparison (** ***P*** **-values)**
Z_3_^–3^		–0.113 ± 0.104	–0.087 ± 0.156	–0.119 ± 0.081	0.553	M vs H: 0.68, M vs E: 0.99, H vs E:0.99
Z_3_^–1^		–0.016 ± 0.126	0.038 ± 0.168	0.034 ± 0.134	0.298	M vs H: 0.48, M vs E: 0.59, H vs E: 0.99
Z_3_^1^		–0.062 ± 0.115	–0.054 ± 0.169	–0.083 ± 0.122	0.722	M vs H: 0.99, M vs E: 0.99, H vs E: 0.99
Z_3_^3^		–0.033 ± 0.110	–0.001 ± 0.109	–0.019 ± 0.088	0.500	M vs H: 0.68, M vs E: 0.99, H vs E: 0.99
Z_4_^–4^		–0.003 ± 0.095	0.021 ± 0.057	0.019 ± 0.045	0.856	M vs H: 0.59, M vs E: 0.70, H vs E: 0.99
Z_4_^–2^		–0.003 ± 0.038	0.015 ± 0.040	–0.018 ± 0.054	0.023	M vs H: 0.40, M vs E: 0.60, H vs E: 0.02
Z_4_^0^		0.191 ± 0.071	0.204 ± 0.085	0.189 ± 0.069	0.728	M vs H: 0.99, M vs E: 0.99, H vs E: 0.99
Z_4_^2^		–0.032 ± 0.041	–0.053 ± 0.059	–0.040 ± 0.045	0.280	M vs H: 0.35, M vs E: 0.99, H vs E: 0.94
Z_4_^4^		–0.031 ± 0.050	–0.044 ± 0.057	–0.030 ± 0.048	0.534	M vs H: 0.99, M vs E: 0.99, H vs E: 0.95
Z_5_^–5^		–0.016 ± 0.027	–0.009 ± 0.040	–0.019 ± 0.027	0.444	M vs H: 0.99, M vs E: 0.99, H vs E: 0.63
Z_5_^–3^		0.007 ± 0.030	0.005 ± 0.023	–0.002 ± 0.018	0.360	M vs H: 0.99, M vs E: 0.52, H vs E: 0.87
Z_5_^–1^		0.002 ± 0.030	0.006 ± 0.030	0.004 ± 0.024	0.596	M vs H: 0.99, M vs E: 0.99, H vs E: 0.99
Z_5_^1^		–0.015 ± 0.016	–0.010 ± 0.024	–0.015 ± 0.015	0.417	M vs H: 0.74, M vs E: 0.99, H vs E: 0.78
Z_5_^3^		–0.005 ± 0.022	–0.002 ± 0.018	–0.001 ± 0.014	0.746	M vs H: 0.99, M vs E: 0.99, H vs E: 0.99
Z_5_^5^		0.006 ± 0.038	–0.008 ± 0.041	0.007 ± 0.051	0.356	M vs H: 0.70, M vs E: 0.99, H vs E: 0.59
RMS	HOAs	0.355 ± 0.073	0.400 ± 0.113	0.348 ± 0.067	0.102	M vs H: 0.16, M vs E: 0.99, H vs E: 0.09
	3 ${}^{\text{rd}}$ Order	0.243 ± 0.098	0.298 ± 0.116	0.250 ± 0.077	0.227	M vs H: 0.12, M vs E: 0.99, H vs E: 0.21
	4 ${}^{\text{th}}$ Order	0.227 ± 0.078	0.241 ± 0.086	0.217 ± 0.073	0.537	M vs H: 0.99, M vs E: 0.99, H vs E: 0.81
	5 ${}^{\text{th}}$ Order	0.068 ± 0.023	0.069 ± 0.034	0.063 ± 0.035	0.323	M vs H: 0.99, M vs E: 0.99, H vs E: 0.99
	T-coma	0.164 ± 0.083	0.228 ± 0.097	0.183 ± 0.086	0.029	M vs H: 0.03, M vs E: 0.99, H vs E:0.19
	T-trefoil	0.172 ± 0090	0.179 ± 0.109	0.158 ± 0.063	0.676	M vs H: 0.99, M vs E: 0.99, H vs E:0.99
RMS, root mean square; HOAs, higher order aberrations; T-coma, Z_3_^1^, Z_3_^−1^, Z_5_^1^, Z_5_^−1^; Trefoil, Z_3_^3^, Z_3_^−3^, Z_5_^3^, Z_5_^−3^; M, myopia; H, hyperopia; E, emmetropia.						

**Table 3:** Ocular aberration coefficients and RMS for refractive error groups

**Zernike term**		**Myopia**	**Hyperopia**	**Emmetropia**	***P*** **-value**	**Pairwise comparison (** ***P*** **-values)**
Z_3_^–3^		0.063 ± 0.102	0.051 ± 0.095	0.057 ± 0.065	0.872	M vs H: 0.99, M vs E: 0.99, H vs E: 0.99
Z_3_^–1^		–0.026 ± 0.088	–0.040 ± 0.098	–0.045 ± 0.077	0.711	M vs H: 0.99, M vs E: 0.99, H vs E: 0.99
Z_3_^1^		–0.014 ± 0.081	0.028 ± 0.090	0.000 ± 0.068	0.142	M vs H: 0.99, M vs E: 0.16, H vs E: 0.61
Z_3_^3^		0.002 ± 0.086	–0.002 ± 0.063	–0.003 ± 0.061	0.963	M vs H: 0.99, M vs E: 0.99, H vs E: 0.99
Z_4_^–4^		–0.011 ± 0.026	–0.013 ± 0.033	–0.009 ± 0.026	0.966	M vs H: 0.99, M vs E: 0.99, H vs E: 0.99
Z_4_^–2^		–0.003 ± 0.024	–0.001 ± 0.022	–0.003 ± 0.018	0.662	M vs H: 0.99, M vs E: 0.99, H vs E: 0.99
Z_4_^0^		–0.025 ± 0.061	–0.065 ± 0.071	–0.047 ± 0.074	0.097	M vs H: 0.10, M vs E: 0.69, H vs E: 0.99
Z_4_^2^		0.009 ± 0.031	–0.008 ± 0.036	0.000 ± 0.040	0.211	M vs H: 0.24, M vs E: 0.99, H vs E: 0.99
Z_4_^4^		–0.001 ± 0.035	0.001 ± 0.031	0.001 ± 0.026	0.748	M vs H: 0.99, M vs E: 0.99, H vs E: 0.99
Z_5_^–5^		0.006 ± 0.016	0.000 ± 0.021	–0.002 ± 0.022	0.147	M vs H: 0.83, M vs E: 0.44, H vs E: 0.99
Z_5_^–3^		–0.012 ± 0.015	–0.006 ± 0.020	–0.004 ± 0.014	0.096	M vs H: 0.44, M vs E: 0.23, H vs E: 0.99
Z_5_^–1^		0.004 ± 0.013	0.003 ± 0.021	0.003 ± 0.014	0.959	M vs H: 0.99, M vs E: 0.99, H vs E: 0.99
Z_5_^1^		0.002 ± 0.009	–0.001 ± 0.016	–0.002 ± 0.015	0.411	M vs H: 0.99, M vs E: 0.63, H vs E: 0.99
Z_5_^3^		0.003 ± 0.009	–0.004 ± 0.015	–0.003 ± 0.017	0.157	M vs H: 0.16, M vs E: 0.24, H vs E: 0.99
Z_5_^5^		0.000 ± 0.015	0.002 ± 0.025	0.003 ± 0.017	0.835	M vs H: 0.99, M vs E: 0.99, H vs E: 0.99
RMS	HOAs	0.199 ± 0.065	0.210 ± 0.077	0.178 ± 0.062	0.214	M vs H: 0.99, M vs E: 0.74, H vs E: 0.26
	3 ${}^{\text{rd}}$ Order	0.179 ± 0.065	0.169 ± 0.078	0.142 ± 0.056	0.055	M vs H: 0.99, M vs E: 0.12, H vs E: 0.37
	4 ${}^{\text{th}}$ Order	0.080 ± 0.037	0.104 ± 0.047	0.093 ± 0.048	0.150	M vs H: 0.13, M vs E: 0.87, H vs E: 0.97
	5 ${}^{\text{th}}$ Order	0.032 ± 0.016	0.045 ± 0.018	0.036 ± 0.020	0.002	M vs H: 0.02, M vs E: 0.99, H vs E: 0.14
	T-coma	0.110 ± 0.055	0.125 ± 0.068	0.101 ± 0.050	0.281	M vs H: 0.99, M vs E: 0.99, H vs E: 0.35
	T-trefoil	0.130 ± 0.071	0.113 ± 0.056	0.096 ± 0.048	0.236	M vs H: 0.82, M vs E: 0.10, H vs E: 0.88
RMS, root mean square; HOAs, higher order aberrations; Tcoma, Z_3_^1^, Z_3_^−1^, Z_5_^1^, Z_5_^−1^; Trefoil, Z_3_^3^, Z_3_^−3^, Z_5_^3^, Z_5_^−3^; M, myopia; H, hyperopia; E, emmetropia.						

##  DISCUSSION

The eye is a complex optical system that can be affected by intrinsic ocular and corneal aberrations, which result in degradation of retinal image quality. The present investigation revealed differences in ocular and corneal aberrations across the three refractive errors, notably higher ocular RMS of the fifth-order, corneal RMS of total coma, and Z_4_^–2^ values in hyperopic eyes.

The results of our investigation showed that hyperopic and myopic eyes exhibited the highest and lowest corneal RMS of T-coma, respectively. Our findings are consistent with those of Llorente et al,^[6]^ who reported that corneal RMS of T-coma was higher by 0.15 µm in hyperopic eyes. The corneal RMS of the third-order was found to be highest in hyperopic eyes, followed by emmetropic eyes and myopic eyes. The observed discrepancies in corneal aberrations among different refraction groups may be attributed to variations in ocular biometric parameters, particularly those related to the cornea. These parameters include corneal curvature, thickness, and shape, all of which play a crucial role in determining the final visual outcome.^[22]^ Additionally, the ocular RMS of the fifth-order displayed a significant difference among the refractive groups.

This study identified a lower ocular RMS of HOAs (0.196 ± 0.069 µm) than that reported by Hashemi et al (0.306 ± 0.16 µm) in the Iranian population.^[23]^ The difference may be attributable to the higher average age of participants in the latter study. On the other hand, our findings are in parallel with the study by Salmon and de Pol,^[24]^ indicating higher ocular third-order aberrations (0.153 ± 0.08 µm) and fourth-order aberrations (0.090 ± 0.47 µm) compared to fifth-order aberrations (0.030 ± 0.02 µm) and sixth-order aberrations (0.02 ± 0.01 µm). Furthermore, our analysis revealed a higher magnitude of ocular coma compared to trefoil in the emmetropic and hyperopic eyes, which is consistent with the results of previous studies.^[5,12,23]^ In line with previous studies, we observed a positive corneal spherical aberration (Z_4_^0^), with no discernible differences among the refractive error groups.^[15,25]^ The results of a relevant study^[26]^ indicated that this similarity could be explained by the lack of significant differences in corneal radius of curvature, asphericity, and Q values.

Several studies have explored potential differences in aberrations across various refractive errors, yielding diverse outcomes. However, similar to some other investigations, the present study did not identify a significant relationship between HOAs and refractive error. Carkeet et al^[13]^ and other investigations^[25,26,27,28]^ demonstrated no substantial variation in HOAs among emmetropes (0.186 ± 0.06 µm), hyperopes (0.184 ± 0.07 µm), and myopes (0.177 ± 0.07 µm). None of these studies identified a significant association between refractive error and HOAs. Martinez et al^[29]^ and Llorente et al^[6]^ concluded that hyperopes displayed a higher incidence of HOAs compared to myopes. In contrast, Kirwan et al^[30]^ reported lower HOAs in hyperopic eyes (0.357 ± 0.13 µm) compared to myopic eyes (0.462 ± 0.10 µm). Additionally, He et al found that individuals with myopia exhibited a higher rate of HOAs (1.26 ± 0.99 µm) than those with emmetropia (0.88 ± 0.28 µm)^5^. Furthermore, Yazar et al documented a higher rate of HOAs in myopia (0.723 ± 0.38 µm) than in emmetropia (0.559 ± 0.26 µm).^[12]^

Lower CF for RMS of HOAs is observed in individuals with emmetropia, hyperopia, and myopia, compared with findings from a previous study.^[15]^ Variations in age and MSE between these studies may account for this discrepancy. Age can affect visual function, as the aging process is often associated with changes in the eye's optical properties, including decreased CF.^[1]^

Discrepancies in findings across studies can be attributed to several factors, including differences in methodology, measurement devices, the use or non-use of cycloplegic-mydriatic drops, and study population demographics.^[12,13,23]^

The study was strengthened by including healthy participants with emmetropia, myopia, and hyperopia, and by an age range of 18-35 years, thereby eliminating the effect of presbyopia. Furthermore, given that astigmatism can affect the measurement of aberrations, we assessed corneal and ocular aberrations independently in the presence of low astigmatism in the participants, thereby enhancing the precision of the comparison among the different refraction groups. These findings also have important clinical implications. The significantly higher RMS values of corneal coma and third-order aberrations in hyperopic eyes underscore the need for personalized approaches in refractive surgery, such as customized ablation profiles, to optimize visual quality and reduce postoperative symptoms, including halos and glare. Additionally, the absence of significant differences in ocular aberrations and CFs across refractive groups implies the relatively uniform capacity of internal optics to compensate for corneal aberrations. This insight may help clinicians better anticipate visual outcomes and refine wavefront-guided correction strategies for patients with different refractive errors.

This study has several limitations that should be acknowledged. Although the sample size was calculated to ensure adequate statistical power based on primary outcome measures, it may still be insufficient to detect smaller or subgroup-specific differences in all aberration parameters across refractive groups. A larger and more diverse sample could further enhance the generalizability of the findings. Additionally, although efforts were made to control for confounding variables—by maintaining a consistent pupil diameter and conducting measurements under scotopic conditions—residual variability due to individual ocular anatomy and tear film stability may have influenced the results. Lastly, the potential effects of accommodation or lens position were not assessed, which could particularly impact internal aberrations in younger participants.

In summary, this study demonstrated that hyperopic eyes exhibited significantly higher corneal RMS values for third-order aberrations and coma, compared to emmetropic and myopic eyes. In contrast, no significant differences were observed in ocular HOAs or in the CF among the three refractive groups. These findings suggest that although corneal aberration profiles vary with refractive status—particularly in hyperopia—the internal optics of the eye appear to compensate for these aberrations in a relatively consistent manner across all refractive errors. The results emphasize the importance of incorporating individual aberration profiles, especially corneal ones, into preoperative planning for refractive surgery to improve visual outcomes and minimize postoperative visual complications.

##  Financial Support and Sponsorship

This work was supported by the Refractive Errors Research Center as well as the Deputy of Research at Mashhad University of Medical Sciences, Iran (Grant number: 4011758).

##  Conflicts of Interest

None.
